# Effect of empagliflozin with or without carvedilol on tricuspid regurgitation and right ventricular remodeling: PROVE trial

**DOI:** 10.1186/s44348-026-00095-4

**Published:** 2026-09-14

**Authors:** Ga Yun Kim, So-Min Lim, Dong Hyun Yang, Jong Eun Lee, Sahmin Lee, Sung-Ji Park, In-Chang Hwang, Yeonyee Elizabeth Yoon, Chi Young Shim, Hyung-Kwan Kim, Sung-Cheol Yun, Jong-Min Song, Duk-Hyun Kang

**Affiliations:** 1 Division of Cardiology, Asan Medical Center, College of Medicine, University of Ulsan, Seoul, Korea; 2 Division of Radiology, Asan Medical Center, College of Medicine, University of Ulsan, Seoul, Korea; 3 Division of Clinical Epidemiology and Biostatistics, Asan Medical Center, College of Medicine, University of Ulsan, Seoul, Korea; 4 Division of Cardiology, Yongin Severance Hospital, Yongin, Korea; 5 Division of Cardiology, Samsung Medical Center, Seoul, Korea; 6 Division of Cardiology, Seoul National University Bundang Hospital, Seongnam, Korea; 7 Division of Cardiology, Severance Medical Center, Seoul, Korea; 8 Division of Cardiology, Seoul National University Hospital, Seoul, Korea

**Keywords:** Tricuspid regurgitation, Multimodality imaging, Doppler echocardiography, Sodium-glucose cotransporter 2 inhibitor

## Abstract

**Background:**

Functional tricuspid regurgitation (TR) is associated with adverse outcomes. As TR, right ventricular (RV) remodeling, and worsening TR form a vicious cycle, therapies that reverse RV remodeling and reduce TR are needed. We examined whether sustained-release carvedilol (carvedilol SR), empagliflozin, or their combination reduces RV volumes and TR severity in patients with significant functional TR.

**Methods:**

In this randomized trial with a 2 × 2 factorial design, 56 patients were assigned to carvedilol SR plus empagliflozin, carvedilol SR, empagliflozin, or placebo. The primary end point was the change in RV end-systolic volume index from baseline to 12 months. Secondary end points included changes in RV end-diastolic volume index, RV ejection fraction, vena contracta width (VCW), effective regurgitant orifice area (EROA), and regurgitant volume. Cardiac magnetic resonance imaging assessed RV volumes, and echocardiography evaluated TR severity.

**Results:**

Baseline characteristics were well balanced between treatment groups (mean age 71 ± 7 years; men 55%; diabetes 14%; atrial fibrillation 89%). Empagliflozin did not change RV volumes or RV ejection fraction. However, empagliflozin reduced VCW (1.45 mm; 95% confidence interval [CI], 0.69–2.20), EROA (0.09 cm^2^; 95% CI, 0.02–0.17), and regurgitant volume (10.7 mL; 95% CI, 4.2–17.2) compared with placebo. Carvedilol SR showed no significant effect on primary or secondary end points. Compared with placebo, carvedilol SR plus empagliflozin reduced VCW (− 0.78 ± 1.15 mm vs. 0.56 ± 1.45 mm, P = 0.03) but did not change other outcomes.

**Conclusions:**

Carvedilol SR, empagliflozin, or their combination showed no significant reduction in RV volumes, but empagliflozin significantly reduced TR severity in patients with functional TR.

**Trial registration:**

ClinicalTrials.gov identifier: NCT04345796 (PROVE trial).

## Background

The presence of significant tricuspid regurgitation (TR) is associated with substantial morbidity and poor long-term survival [1–3]. In patients with symptomatic, severe primary TR, tricuspid valve surgery can be beneficial, but management of secondary TR accounting for most cases of severe TR remains controversial [3–5]. Secondary, functional TR is commonly observed in patients with atrial fibrillation, heart failure (HF), and left-sided valve disease, and management of the underlying etiologies may decrease the severity of functional TR [5]. However, guideline-directed medical therapy (GDMT) for HF often fails to improve TR, and diuretics are provided to relieve systemic congestive symptoms associated with TR [5–7]. Functional TR may progress over time, because TR causes dilatation of the right ventricle (RV), which can subsequently result in further enlargement of tricuspid annulus and tethering of the leaflets, finally leading to deterioration of TR [8, 9]. As a vicious cycle of TR, RV remodeling, and worsening TR results in RV failure, which is closely related to poor prognosis, therapies that induce reversal of RV remodeling and consequently reduce TR may improve clinical outcomes. However, there have been no proven medical therapies for adverse remodeling of the RV and secondary TR [5].

Sodium-glucose cotransporter 2 (SGLT2) inhibitors reduce the risk of cardiovascular death and hospitalization for HF [10–13], and are included in the GDMT for HF [6]. SGLT2 inhibitors may also be a therapeutic option for RV failure because of their diuretic and nephroprotective effects, but their effects on RV remodeling and functional TR are unknown. Activation of the sympathetic nervous system contributes to progression of right- and left-sided HF via its effects on adverse ventricular remodeling [14]. While beta-blockers are a standard medical therapy for left ventricle (LV) failure [6], their use in managing RV failure is not well established. Therefore, the PROVE trial (Pharmacologic Reduction of Right Ventricular Enlargement) was designed to examine whether treatment with a SGLT2 inhibitor; empagliflozin, the beta-blocker; sustained-release carvedilol (carvedilol SR), or their combination would reduce RV volumes, compared with placebo. Furthermore, we also sought to evaluate the therapeutic efficacy of empagliflozin, carvedilol SR, or their combination on functional TR.

## Methods

### Study design

PROVE was a prospective, multicenter, randomized, investigator-initiated trial with a 2-by-2 factorial design. The trial was conducted at five centers in Korea. In the first randomized comparison, we examined the effect of treatment with carvedilol SR at a daily dose of 16 mg, as compared with placebo, on the changes in RV volumes (primary outcome) and severity of TR (secondary outcome). In the second randomized comparison, we compared primary and secondary outcomes of patients assigned to GDMT plus empagliflozin 10 mg per day with those assigned to GDMT alone. We also assessed the effects of carvedilol SR plus empagliflozin, as compared with placebo. The study protocol was designed by the principal investigator and approved by the institutional review board at each participating center. The first draft of the manuscript was prepared by the first author, and was reviewed and edited by all the authors. All the authors made the decision to submit the manuscript for publication and vouch for the fidelity of this report to the protocol and for the accuracy and completeness of the data and the analyses.

### Patient selection

Patients were eligible for enrollment if they were at least 20 years of age; had stable HF with New York Heart Association (NYHA) class II or III symptoms, with or without diabetes; had an LV ejection fraction (LVEF) ≥ 50%; and had significant functional TR, defined as a vena contracta width ≥ 0.7 cm or central jet area > 10 cm^2^, persisting for > 6 months despite GDMT. Patients were required to receive a stable, optimized GDMT for at least 4 weeks before enrollment.

Exclusion criteria included symptomatic hypotension, a systolic blood pressure < 90 mmHg, bradycardia with heart rate < 50 beats/min or 2nd or 3rd degree atrioventricular block, LVEF < 50%, a glycated hemoglobin level > 10.5%, an estimated glomerular filtration rate < 30 mL/min per 1.73 m^2^, or a history of ketoacidosis. Patients were also excluded from the trial if they had any evidence of structural tricuspid valve disease; previous tricuspid valve intervention; significant left-sided valve disease, severe pulmonary hypertension; NYHA IV symptoms; previous hospitalization within 6 weeks; previous history of acute coronary syndrome, stroke, percutaneous coronary intervention, or cardiovascular surgery within 3 months; an indication for cardiac resynchronization therapy; a plan of coronary revascularization, tricuspid valve intervention, or heart transplantation during the trial. All the participants provided written informed consent.

### Study procedures

Eligible patients entered a run-in phase lasting 4 weeks, during which they received placebo alone. Patients who had at least 80% adherence to placebo were randomly assigned to receive GDMT for HF alone or GDMT plus 10 mg of empagliflozin, carvedilol SR or matching placebo, with the use of a computerized randomization system involving concealed study-group assignments. The randomization was stratified according to presence or absence of diabetes and cardiac rhythm. The interactive web response system assigned a randomization number to each patient, which linked the patient to a treatment group, to keep the study information confidential and specified a unique medication number for the study drug to be dispensed. Carvedilol SR and the matching placebo were identical in appearance. All other treatments, including GDMT, were continued. Patients were treated for 12 months and evaluated every 4 weeks during the first 2 months of therapy and every 2 to 6 months thereafter.

Cardiac magnetic resonance (CMR) imaging and echocardiographic evaluation were performed at randomization and at the 12-month follow-up visit. CMR was conducted using the latest 3.0 T magnetic resonance imaging scanner equipped with phased-array cardiac coils at participating centers, all of which had been approved by a central CMR core laboratory for their equipment and compliance with the specified acquisition protocols. CMR data collected at each site were anonymized and electronically transferred to a central server (AiCROsystem, Asan Image Metrics) for image storage and unbiased, independent image analysis [15]. Cine CMR images were analyzed using a standardized technique with commercially available software (CMR 42, Circle Cardiovascular Imaging) [16]. The short-axis cine images, spanning from the base to the apex of the RV, were measured by an investigator (DHY) who was blinded to patient data, treatment allocation, study sequence, and any previous measurements. Experienced sonographers who were certified by the echocardiography core laboratory and dedicated to this trial performed a standard two-dimensional and Doppler echocardiographic examination on all patients using an iE33 echocardiography system (Philips). Echocardiographic studies were stored digitally in Digital Imaging and Communications in Medicine format and transferred to the echocardiography core laboratory, where measurements were performed using an offline digital review workstation by an investigator (DHK) who was masked to all the information related to the trial. The end-systolic volume, end-diastolic volume, and EF of the LV and end-systolic volume of the left atrium were calculated with the biplane Simpson method [17]. For vena contracta measurement, the ultrasound beam was oriented as perpendicular to the regurgitant flow as possible, and the width of the narrowest portion of TR jet flow at or immediately downstream of the regurgitant orifice was measured in a zoomed view. Vena contracta width was measured on RV modified apical 4-chamber and RV inflow views, and the measurements were averaged [18, 19]. Effective regurgitant orifice area (EROA) was determined by dividing the regurgitant flow rate, calculated as 2πr^2^ × aliasing velocity, where r is the proximal isovelocity surface area (PISA) radius, by peak TR velocity [19]. The regurgitant volume was estimated as EROA multiplied by the velocity time integral of the TR jet.

### Outcomes

The primary end point was the change in RV end-systolic volume index from baseline to the 12-month follow-up. Secondary end points included changes in RV end-diastolic volume index, RV ejection fraction (RVEF), vena contracta width, EROA of TR, and regurgitant volume.

### Statistical analysis

Based on our previous study [20], we assumed a common baseline mean RV end-systolic volume index of 88.5 mL/m^2^, a common standard deviation of 30.1 mL/m^2^. Given these assumptions, we calculated that a sample size of 164 patients randomly assigned to two groups would provide 80% power to detect a difference of 15% in the RV end-systolic volume index between the carvedilol SR and placebo groups, using a two-sided t-test with an alpha level of 0.05. The study plan was to randomize 180 patients, allowing for a drop-out rate of 10%. Because the COVID-19 pandemic seriously affected enrollment of study patients, resulting in a lower rate of patient enrollment than expected, the sponsor had a logistic problem in providing study drugs beyond the planned timeline. On the basis of a limited supply of study drug, the executive committee decided to stop enrollment in December 2023 without knowledge of any trial data and the actual number of study patients was 56.

The primary analysis for each treatment comparison was based on all participants who had undergone randomization. If no interactions were observed between the main effects of carvedilol SR and empagliflozin, each individual comparison was to be performed. Analyses followed the intention-to-treat principle, with all randomized patients analyzed in their originally assigned groups. Baseline clinical and echocardiographic characteristics were compared in the two treatment groups using Student’s t-test or the Mann–Whitney U test for continuous variables and the chi-square test or Fisher’s exact test for categorical variables as appropriate. The null hypothesis was that there would be no difference between the treatment groups regarding the change in RV end-systolic volume index from baseline to 12-month follow-up, and an intention-to-treat analysis tested this hypothesis. For the analyses of primary and secondary end points, we used the t-test for differences between groups as described in the protocol. Estimated between-group differences are presented with 95% confidence interval (CI). All reported P-values were two-sided, and P < 0.05 was considered statistically significant. SAS ver. 9.1 (SAS Institute) and R ver. 3.3.1 (R Foundation for Statistical Computing) were used for statistical analyses.

## Results

### Patient characteristics

Between January 2021 and July 2023, a total of 56 patients completed the run-in phase; 13 patients were randomly assigned to receive carvedilol SR plus empagliflozin, 14 to receive carvedilol SR, 14 to receive empagliflozin, and 15 to receive placebo. CMR and echocardiography were performed at a median interval of 28 days (interquartile range, 0 to 37.5 days) across the baseline and 12-month assessments, with both studies performed on the same day in 38% of assessments. The treatment groups were generally well balanced with regard to baseline clinical, CMR, and echocardiographic characteristics, except for age and RV end-systolic volume index **(**Table 1**)**. The mean (± SD) age of the patients was 70.9 ± 6.8 years and 31 (55%) were men, 8 (14%) had diabetes, 54 (96%) were in NYHA functional class II and 2 (4%) in NYHA class III, and atrial fibrillation was present in 50 (89%). The mean LVEF was 60 ± 7%, the mean RV end-systolic volume index was 59 ± 22 mL/m^2^, the mean RV end-diastolic volume index was 113 ± 35 mL/m^2^, and the mean RVEF was 48 ± 9%. The mean vena contracta width was 8.6 ± 2.2 mm, the mean EROA of TR was 0.52 ± 0.25 cm^2^, and the mean regurgitant volume was 44 ± 19 mL. Most patients received GDMT, and diuretics were used in 39 (70%) patients and beta-blockers in 30 (54%).

**Table 1:** Baseline characteristics of the patients according to treatment group

Characteristic	Carvedilol SR plus empagliflozin (*n* = 13)	Carvedilol SR alone (*n* = 14)	Empagliflozin alone (*n* = 14)	Placebo alone (*n* = 15)	*P*-value
Age (yr)	75 ± 5	72 ± 6	70 ± 7	68 ± 7	0.025
Male sex	5 (38.5)	9 (64.3)	6 (42.9)	11 (73.3)	0.187
BMI (kg/m^2^)	24.2 ± 3.3	24.4 ± 3.2	25.4 ± 2.7	25.5 ± 2.6	0.397
Hypertension	9 (69.2)	9 (64.3)	8 (57.1)	9 (60.0)	0.946
Diabetes	2 (15.4)	1 (7.1)	2 (14.3)	3 (20.0)	0.918
Atrial fibrillation	12 (92.3)	14 (100)	11 (78.6)	13 (86.7)	0.385
Chronic kidney disease	3 (23.1)	3 (21.4)	4 (28.6)	1 (6.7)	0.490
Coronary artery disease	2 (15.4)	1 (7.1)	2 (14.3)	5 (33.3)	0.337
NYHA functional class					0.051
II	11 (84.6)	14 (100)	14 (100)	15 (100)	
III	2 (15.4)	0 (0)	0 (0)	0 (0)	
Heart failure medication					
ARNI	1 (7.7)	1 (7.1)	1 (7.1)	1 (6.7)	> 0.999
ACE inhibitor	0 (0)	1 (7.1)	0 (0)	0 (0)	0.732
ARB	5 (38.5)	4 (28.6)	6 (42.9)	10 (66.7)	0.222
Beta-blocker	8 (61.5)	7 (50.0)	6 (42.9)	9 (60.0)	0.762
Diuretic	12 (92.3)	8 (57.1)	9 (64.3)	10 (66.7)	0.194
Aldosterone antagonist	3 (23.1)	3 (21.4)	2 (14.3)	1 (6.7)	0.609
Systolic blood pressure (mmHg)	129 ± 12	119 ± 14	128 ± 15	127 ± 15	0.357
Diastolic blood pressure (mmHg)	76 ± 7	73 ± 7	77 ± 13	73 ± 11	0.634
Heart rate (beats/min)	75 ± 11	78 ± 9	83 ± 12	73 ± 11	0.111
Hemoglobin (g/dL)	13.3 ± 1.5	13.3 ± 1.6	13.2 ± 1.6	14.4 ± 1.6	0.229
Albumin (g/dL)	4.0 ± 0.3	4.0 ± 0.5	4.2 ± 0.3	4.0 ± 0.3	0.125
Creatinine (mg/dL)	0.91 ± 0.26	0.92 ± 0.22	0.95 ± 0.19	0.96 ± 0.19	0.862
Glucose (mg/dL)	123 ± 53	105 ± 23	118 ± 22	113 ± 33	0.270
HbA1c (%)	6.3 ± 1.4	5.7 ± 0.6	6.1 ± 0.5	5.9 ± 0.6	0.109
Cholesterol (mg/dL)	160 ± 26	158 ± 36	156 ± 31	154 ± 36	0.993
LDL cholesterol (mg/dL)	83 ± 30	100 ± 21	77 ± 29	73 ± 25	0.428
NT-proBNP (pg/mL)	1012 ± 419	1289 ± 984	638 ± 413	897 ± 706	0.075
Echocardiographic measure					
Left heart structure and function					
LV end-diastolic dimension (mm)	48.0 ± 6.4	50.5 ± 3.6	45.6 ± 4.8	50.8 ± 6.4	0.042
LV end-systolic dimension (mm)	31.2 ± 7.8	32.9 ± 5.0	29.4 ± 4.6	33.9 ± 5.3	0.096
LVEF (%)	59.9 ± 8.1	59.5 ± 7.1	58.7 ± 6.3	60.5 ± 5.3	0.939
Left atrial volume index (mL/m^2^)	71 ± 32	64 ± 15	54 ± 16	76 ± 38	0.401
E/e´ ratio	10.9 ± 2.7	12.1 ± 8.3	9.6 ± 3.0	13.0 ± 7.0	0.290
Right heart structure and function					
Vena contracta width (mm)	9.20 ± 1.85	7.67 ± 2.07	9.29 ± 2.72	8.47 ± 2.06	0.149
EROA (cm^2^)	0.57 ± 0.22	0.48 ± 0.22	0.55 ± 0.31	0.51 ± 0.27	0.149
Regurgitant volume (mL)	46.9 ± 19.3	40.6 ± 19.4	44.6 ± 23.5	42.3 ± 16.7	0.810
TAPSE (mm)	19.6 ± 3.4	19.1 ± 4.2	19.4 ± 2.6	19.3 ± 3.8	0.998
TR peak velocity (m/sec)	2.7 ± 0.2	2.8 ± 0.3	2.7 ± 0.3	2.8 ± 0.3	0.554
Cardiac magnetic resonance measure					
LV end-diastolic volume index (mL/m^2^)	75.3 ± 12.7	75.6 ± 20.2	78.3 ± 29.4	81.3 ± 21.7	0.890
LV end-systolic volume index (mL/m^2^)	32.4 ± 10.5	34.7 ± 12.4	36.9 ± 27.9	39.5 ± 12.1	0.354
RV end-diastolic volume index (mL/m^2^)	104.1 ± 21.2	103.5 ± 41.0	112.9 ± 31.5	130.5 ± 36.5	0.183
RV end-systolic volume index (mL/m^2^)	52.4 ± 14.3	50.1 ± 22.7	56.0 ± 15.9	75.4 ± 25.9	0.010
RVEF (%)	49.8 ± 7.6	51.0 ± 7.2	50.1 ± 7.3	42.3 ± 9.8	0.089

Values are presented as number (%) or mean ± standard deviation

ACE, angiotensin-converting enzyme; ARB, angiotensin receptor blocker; ARNI, angiotensin receptor-neprilysin inhibitor; BMI, body mass index; carvedilol SR, sustained-release carvedilol; E/e’ ratio, the ratio of early diastolic mitral inflow velocity to early diastolic mitral annular tissue velocity at the septal annulus; EROA, effective regurgitant orifice area; HbA1c, glycated hemoglobin; LDL, low-density lipoprotein; LV, left ventricle; LVEF, left ventricular ejection fraction; NT-proBNP, N-terminal pro-B-type natriuretic peptide; NYHA, New York Heart Association; RV, right ventricle; RVEF, right ventricular ejection fraction; TAPSE, tricuspid annular plane systolic excursion; TR, tricuspid regurgitation

### Follow-up

Among the 13 patients randomly assigned to carvedilol SR plus empagliflozin, 3 patients withdrew from the trial (1 had a serious adverse event of bradycardia) and 10 completed the trial. In the carvedilol SR group (n = 14), 4 patients withdrew from the trial (1 had a serious adverse event of hyperkalemia) and 10 completed the trial. In the empagliflozin group (n = 14), 3 patients withdrew from the trial and 11 completed the trial. Of the 15 patients randomly assigned to placebo, 2 patients withdrew from the trial and 13 completed the trial. No patient underwent cardiac surgery or percutaneous intervention during the trial period. Although the original analysis plan specified an intention-to-treat analysis, the primary analysis included 44 patients who completed the study.

### Outcomes for carvedilol SR as compared with placebo

After 12 months of treatment, the primary end point of change in RV end-systolic volume index was not significantly different between the carvedilol SR and placebo groups (–0.55 ± 9.80 mL/m^2^ vs. –4.75 ± 17.47 mL/m^2^, P = 0.323) **(**Table 2**)**. The change in RV end-diastolic volume index (–0.65 ± 22.90 mL/m^2^ vs. –1.30 ± 27.94 mL/m^2^, P = 0.935) and RVEF (1.14 ± 9.59% vs. 2.62 ± 10.03%, P = 0.624) were similar between the treatment groups. Changes in vena contracta width of TR, EROA, and regurgitant volume also did not differ significantly between the treatment groups.

**Table 2:** Outcomes of carvedilol SR versus placebo

Outcome	Baseline			Follow-up			Change			
	Carvedilol SR (n = 20)	Placebo (n = 24)	P-value	Carvedilol SR (n = 20)	Placebo (n = 24)	*P*-value	Carvedilol SR (n = 20)	Placebo (n = 24)	Difference (95% CI)	P-value
Primary end point										
RV end-systolic volume index (mL/m^2^)	50.5 ± 13.7	69.2 ± 24.0	0.002	49.9 ± 16.6	64.5 ± 22.6	0.021	–0.55 ± 9.80	–4.75 ± 17.47	4.19 (–4.67 to 13.06)	0.323
Secondary end point										
RV end-diastolic volume index (mL/m^2^)	104.2 ± 25.8	127.5 ± 35.2	0.018	103.5 ± 28.9	126.2 ± 42.9	0.051	–0.65 ± 22.9	–1.30 ± 27.94	0.64 (–15.12 to 16.41)	0.935
RVEF (%)	50.9 ± 8.1	45.7 ± 9.8	0.065	52.1 ± 9.0	48.4 ± 8.5	0.167	1.14 ± 9.59	2.62 ± 10.03	–1.47 (–7.48 to 4.53)	0.624
TR VC width (mm)	8.08 ± 1.72	9.23 ± 2.44	0.083	8.04 ± 1.85	9.10 ± 2.79	0.152	–0.04 ± 1.25	–0.12 ± 1.57	0.08 (–0.80 to 0.97)	0.853
TR EROA (cm^2^)	0.52 ± 0.25	0.58 ± 0.29	0.513	0.52 ± 0.27	0.57 ± 0.29	0.583	–0.004 ± 0.14	–0.01 ± 0.11	0.01 (–0.07 to 0.09)	0.859
TR regurgitant volume (mL)	44.1 ± 21.6	47.1 ± 19.7	0.625	44.4 ± 22.4	46.1 ± 20.9	0.800	0.35 ± 14.16	–1.00 ± 9.44	1.35 (–5.98 to 8.68)	0.712

Values are presented as mean ± standard deviation

Carvedilol SR, sustained-release carvedilol; CI, confidence interval; EROA, effective regurgitant orifice area; RV, right ventricle; RVEF, right ventricular ejection fraction; TR, tricuspid regurgitation; VC, vena contracta

### Outcomes for empagliflozin as compared with placebo

**Fig. 1:**
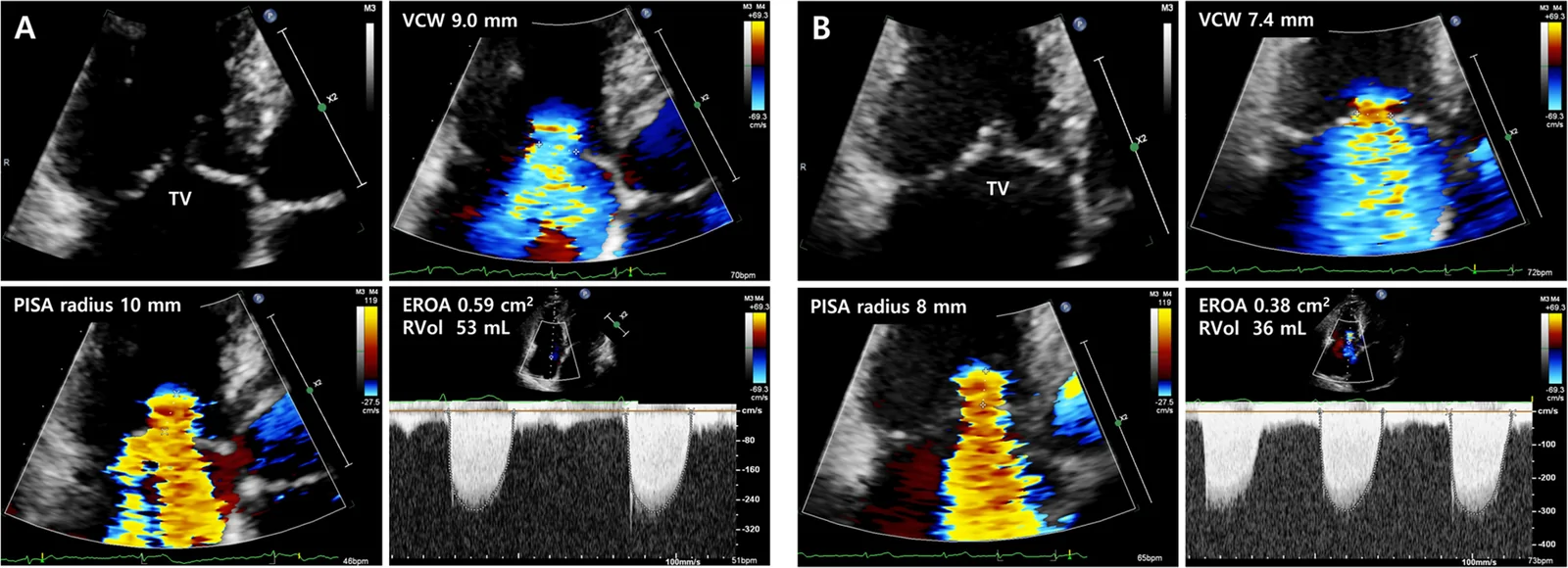
Baseline echocardiography shows tethering of tricuspid valve with severe functional tricuspid regurgitation (TR). Vena contracta width (VCW) was measured, effective regurgitant orifice area (EROA) and regurgitant volume were calculated with proximal isovelocity surface area (PISA) radius, peak velocity and velocity time integral of the TR jet (**A**). Tethering of tricuspid valve was improved and VCW, EROA, and regurgitant volume were significantly decreased after treatment with empagliflozin for 12 months (**B**). EROA, effective regurgitant orifice area; PISA, proximal isovelocity surface area; RVol, regurgitant volume; TV, tricuspid valve; VCW, vena contracta width

The primary end point of change in RV end-systolic volume index did not differ significantly between the empagliflozin and placebo groups (–1.97 ± 16.20 mL/m^2^ vs. –3.64 ± 13.07 mL/m^2^, P = 0.708) (Table 3). There was also no significant difference in the change in RV end-diastolic volume index (–2.72 ± 29.09 mL/m^2^ vs. 0.56 ± 22.28 mL/m^2^, P = 0.675) and RVEF (0.35 ± 9.15% vs. 3.41 ± 10.24%, P = 0.304) between the treatment groups. Regarding secondary end points of TR severity, the changes in vena contracta width (–0.82 ± 1.26 mm vs. 0.62 ± 1.20 mm, P < 0.001), EROA (–0.06 ± 0.15 cm^2^ vs. 0.04 ± 0.07 cm^2^, P = 0.013), and regurgitant volume (–5.86 ± 12.82 mL vs. 4.86 ± 7.77 mL, P = 0.002) were significantly different (Fig. 1). Compared with placebo, empagliflozin significantly reduced vena contracta width by 1.45 mm (95% CI, 0.69–2.20), EROA of TR by 0.09 cm^2^ (95% CI, 0.02–0.17), and regurgitant volume by 10.72 mL (95% CI, 4.23–17.22).

**Table 3:** Outcomes of empagliflozin versus placebo

Outcome	Baseline			Follow-up			Change			
	Empagliflozin (n = 21)	Placebo (n = 23)	P-value	Empagliflozin (n = 21)	Placebo (n = 23)	P-value	Empagliflozin (n = 21)	Placebo (n = 23)	Difference (95% CI)	P-value
Primary end point										
RV end-systolic volume index (mL/m^2^)	56.8 ± 14.8	64.3 ± 26.7	0.254	54.8 ± 20.6	60.6 ± 21.9	0.371	–1.97 ± 16.20	–3.64 ± 13.07	1.67 (–7.25 to 10.59)	0.708
Secondary end point										
RV end-diastolic volume index (mL/m^2^)	113.7 ± 27.1	119.8 ± 38.1	0.546	111.0 ± 42.3	120.4 ± 35.1	0.425	–2.72 ± 29.09	0.56 ± 22.28	–3.28 (–18.97 to 12.40)	0.675
RVEF (%)	49.7 ± 7.5	46.6 ± 10.6	0.276	50.1 ± 8.1	50.0 ± 9.6	0.991	0.35 ± 9.15	3.41 ± 10.24	–3.06 (–8.99 to 2.87)	0.304
TR VC width (mm)	9.47 ± 2.23	8.00 ± 1.95	0.026	8.64 ± 2.42	8.57 ± 2.49	0.926	–0.82 ± 1.26	0.62 ± 1.20	–1.45 (–2.20 to –0.69)	< 0.001
TR EROA (cm^2^)	0.59 ± 0.27	0.51 ± 0.27	0.350	0.54 ± 0.30	0.55 ± 0.27	0.858	–0.06 ± 0.15	0.04 ± 0.07	–0.09 (–0.17 to –0.02)	0.013
TR regurgitant volume (mL)	48.7 ± 21.5	43.0 ± 19.5	0.360	42.9 ± 21.7	47.6 ± 21.3	0.470	–5.86 ± 12.82	4.86 ± 7.77	–10.72 (–17.22 to –4.23)	0.002

Values are presented as mean ± standard deviation

CI, confidence interval; EROA, effective regurgitant orifice area; RV, right ventricle; RVEF, right ventricular ejection fraction; TR, tricuspid regurgitation; VC, vena contracta

### Outcomes for carvedilol SR plus empagliflozin as compared with placebo

Between the carvedilol SR plus empagliflozin and placebo groups, RV end-systolic volume index (–2.80 ± 10.09 mL/m^2^ vs. –7.74 ± 14.28 mL/m^2^, P = 0.364), RV end-diastolic volume index (–4.33 ± 20.90 mL/m^2^ vs. –1.33 ± 20.45 mL/m^2^, P = 0.733), and RVEF (1.36 ± 10.50% vs. 5.31 ± 10.96%, P = 0.392) did not differ significantly (Table 4). There was also no significant difference in the change in EROA (–0.06 ± 0.17 cm^2^ vs. 0.02 ± 0.07 cm^2^, P = 0.129) and regurgitant volume (–6.00 ± 16.24 mL vs. 3.33 ± 7.23 mL, P = 0.118) between the treatment groups, but carvedilol SR plus empagliflozin significantly reduced the vena contracta width by 1.34 mm (95% CI, 0.16–2.52), compared with placebo (P = 0.029).

**Table 4:** Outcomes of carvedilol SR plus empagliflozin versus placebo

Outcome	Baseline			Follow-up			Change			
	Carvedilol SR plus Empagliflozin (n = 10)	Placebo (n = 13)	P-value	Carvedilol SR plus Empagliflozin (n = 10)	Placebo (n = 13)	P-value	Carvedilol SR plus Empagliflozin (n = 10)	Placebo (n = 13)	Difference (95% CI)	P-value
Primary end point										
RV end-systolic volume index (mL/m^2^)	54.1 ± 14.3	77.7 ± 27.1	0.021	51.3 ± 18.2	69.9 ± 21.8	0.041	–2.80 ± 10.09	–7.74 ± 14.28	4.94 (–6.13 to 16.01)	0.364
Secondary end point										
RV end-diastolic volume index (mL/m^2^)	106.4 ± 20.4	133.6 ± 38.2	0.055	102.1 ± 29.4	132.2 ± 35.2	0.040	–4.33 ± 20.90	–1.33 ± 20.45	–3.00 (–21.06 to 15.06)	0.733
RVEF (%)	49.1 ± 8.5	41.9 ± 10.5	0.092	50.5 ± 8.5	47.2 ± 8.8	0.384	1.36 ± 10.50	5.31 ± 10.96	–3.95 (–13.37 to 5.46)	0.392
TR VC width (mm)	8.93 ± 1.52	8.61 ± 2.09	0.686	8.15 ± 2.14	9.12 ± 3.00	0.404	–0.78 ± 1.15	0.56 ± 1.45	–1.34 (–2.52 to –0.16)	0.029
TR EROA (cm^2^)	0.55 ± 0.25	0.53 ± 0.29	0.857	0.49 ± 0.30	0.56 ± 0.29	0.609	–0.06 ± 0.17	0.02 ± 0.07	–0.09 (–0.20 to 0.03)	0.129
TR regurgitant volume (mL)	46.1 ± 21.5	43.8 ± 17.6	0.777	40.1 ± 21.9	46.8 ± 20.4	0.470	–6.00 ± 16.24	3.33 ± 7.23	–9.33 (–20.18 to 1.51)	0.118

Values are presented as mean ± standard deviation

Carvedilol SR, sustained-release carvedilol; CI, confidence interval; EROA, effective regurgitant orifice area; RV, right ventricle; RVEF, right ventricular ejection fraction; TR, tricuspid regurgitation; VC, vena contracta

## Discussion

The PROVE trial showed no significant reduction in RV volumes with carvedilol SR, empagliflozin, or their combination compared with placebo. Although no significant change in TR severity was observed with carvedilol SR, empagliflozin significantly reduced vena contracta width of TR, EROA and regurgitant volume.

In the present study, different imaging modalities were used to assess RV volumes and TR severity: CMR for RV volumes and echocardiography for TR. Because CMR is considered the gold standard for quantitative assessment of RV volumes due to the complex shape of the RV and excellent endocardial resolution [19, 21], CMR was used for volumetric analysis of the RV in this trial. CMR is also feasible for quantitative evaluation of TR; however, atrial fibrillation is often associated with functional TR and may pose a challenge for CMR quantification of TR, since most CMR acquisitions are obtained over multiple cardiac cycles [19]. In contrast, echocardiographic assessment of TR has been the standard approach to determine the severity of TR [5, 19], and recent trials involving transcatheter repair and transcatheter valve replacement for TR used echocardiography as the primary imaging modality [22, 23]. Functional TR is common and usually silent in patients with HF [21], and echocardiography is highly useful for evaluating functional TR because of its accessibility, real-time imaging capabilities, and cost-effectiveness, making it the diagnostic modality of choice for evaluation of TR [21, 24]. We set the change in severity of TR as a secondary outcome and included measurement of vena contracta width, EROA, and regurgitant volume in secondary end points, because there was less experience with quantitation of TR severity with PISA compared with mitral regurgitation (MR) [25, 26]. No single Doppler measurement or parameter is precise enough to quantify TR severity; however, TR severity can be determined with high probability, when multiple parameters are concordant [19]. In the present study, the SGLT2 inhibitor, empagliflozin consistently reduced multiple parameters of TR severity significantly.

The current guidelines provide no class I recommendation for medical management of functional TR, and diuretic therapy alone has a class IIa recommendation for right-sided HF attributable to severe TR [5]. Functional TR and MR are common in HF with preserved EF (HFpEF), and relate to underlying atrial myopathy of the left atrium and/or right atrium [21, 27]. While use of beta-blockers is not recommended for HFpEF, SGLT2 inhibitors can be beneficial in HFpEF based on remarkable outcomes of recent clinical trials [6, 13, 14], and may be considered for pharmacological management of functional TR and MR associated with HFpEF. In the EFFORT trial (Ertugliflozin for Functional Mitral Regurgitation), we previously demonstrated that compared with placebo, the SGLT2 inhibitor ertugliflozin significantly reduced functional MR associated with HF with mildly reduced EF, although there were no significant between-group differences regarding the changes in LV volume indices and LVEF [26]. The PROVE trial presently showed that the SGLT2 inhibitor empagliflozin showed no significant reduction in RV volumes compared with placebo, but improved functional TR in patients with HFpEF. This study could not determine the mechanism underlying the effect of empagliflozin on TR severity, and further studies are needed to evaluate the way in which empagliflozin works for the reduction of functional TR.

Management of severe functional TR should be performed by a multidisciplinary team and optimization of medical therapy is required before consideration of transcatheter therapies for TR [3, 21]. Despite current recommendations of SGLT2 inhibitors for HFpEF, SGLT2 inhibitors are rarely used in patients with functional TR and HFpEF, because their effects on functional TR are uncertain [22]. In this regard, the EVENT trial (Enavogliflozin Outcome Trial in Functional Tricuspid Regurgitation, NCT06027307) will test the hypothesis that the SGLT2 inhibitor enavogliflozin, compared with placebo, will improve clinical and echocardiographic outcome in functional TR associated with HFpEF. This ongoing trial, to be completed in 2027, will document the impact of enavogliflozin versus matching placebo on the occurrence of a composite of cardiovascular events or worsening of TR in 540 patients with functional TR and HFpEF.

## Limitations

Because enrollment of trial patients was affected by the COVID-19 pandemic, the present trial did not reach the calculated sample size to provide 80% power to detect a difference of 15% in the RV end-systolic volume index between the treatment groups, thereby resulting in an underpowered study. The primary analysis did not follow the intention-to-treat principle and instead included only patients who completed the study, owing to the small number of patients in this trial. In this trial, matching placebo for empagliflozin was not provided. However, the assessors were blinded to patient data, treatment allocation, study sequence, and previous measurements in each patient. The daily dose of carvedilol SR used in this trial was lower than the target dose recommended for heart failure with reduced ejection fraction [6]. Our trial enrolled patients with HFpEF, for whom no established target dose of carvedilol exists and the use of beta-blockers for managing RV failure is not well established. Therefore, the lack of efficacy of carvedilol SR may be dose-related rather than reflecting a true class effect of beta-blockers. Because our trial included patients with an LVEF ≥ 50%, the results of our study may not be applicable to patients with LVEF < 50%. Finally, our small trial is a mechanistic study, not an outcome trial, and the statistical analyses were performed according to the prespecified study protocol without formal adjustment for multiple comparisons.

## Conclusion

Among patients with significant functional TR, carvedilol SR, empagliflozin, or their combination showed no significant reduction in RV volumes compared with placebo. However, empagliflozin significantly reduced functional TR. A future outcome trial will determine the efficacy of SGLT2 inhibitors on functional TR secondary to HFpEF.

## Data Availability

The data collected for this study will not be made available to others.

## Abbreviations

Carvedilol SR: Sustained-release carvedilol
CI: Confidence interval
CMR: Cardiac magnetic resonance
EROA: Effective regurgitant orifice area
GDMT: Guideline-directed medical therapy
HF: Heart failure
LV: Left ventricle
LVEF: Left ventricular ejection fraction
MR: Mitral regurgitation
NYHA: New York Heart Association
PISA: Proximal isovelocity surface area
RV: Right ventricle
RVEF: Right ventricular ejection fraction
SGLT2: Sodium-glucose cotransporter 2
TR: Tricuspid regurgitation

## References

[1] Wang N, Fulcher J, Abeysuriya N, McGrady M, Wilcox I, Celermajer D, et al. Tricuspid regurgitation is associated with increased mortality independent of pulmonary pressures and right heart failure: a systematic review and meta-analysis. Eur Heart J. 2019;40:476–84. 30351406 10.1093/eurheartj/ehy641

[2] Nath J, Foster E, Heidenreich PA. Impact of tricuspid regurgitation on long-term survival. J Am Coll Cardiol. 2004;43:405–9. 15013122 10.1016/j.jacc.2003.09.036

[3] Hahn RT. Tricuspid regurgitation. N Engl J Med. 2023;388:1876–91. 37195943 10.1056/NEJMra2216709

[4] Vieitez JM, Monteagudo JM, Mahia P, Perez L, Lopez T, Marco I, et al. New insights of tricuspid regurgitation: a large-scale prospective cohort study. Eur Heart J Cardiovasc Imaging. 2021;22:196–202. 32783057 10.1093/ehjci/jeaa205

[5] Otto CM, Nishimura RA, Bonow RO, Carabello BA, Erwin JP, et al. 2020 ACC/AHA guideline for the management of patients with valvular heart disease: a report of the American College of Cardiology/American Heart Association Joint Committee on Clinical Practice Guidelines. J Am Coll Cardiol. 2020;77:e25-197. 33342586 10.1016/j.jacc.2020.11.018

[6] Heidenreich PA, Bozkurt B, Aguilar D, Allen LA, Byun JJ, Colvin MM, et al. 2022 AHA/ACC/HFSA guideline for the management of heart failure: a report of the American College of Cardiology/American Heart Association Joint Committee on Clinical Practice Guidelines. Circulation. 2022;145:e895-1032. 35363499 10.1161/CIR.0000000000001063

[7] Hahn RT, Brener MI, Cox ZL, Pinney S, Lindenfeld J. Tricuspid regurgitation management for heart failure. JACC Heart Fail. 2023;11:1084–102. 37611990 10.1016/j.jchf.2023.07.020

[8] Topilsky Y, Nkomo VT, Vatury O, Michelena HI, Letourneau T, Suri RM, et al. Clinical outcome of isolated tricuspid regurgitation. JACC Cardiovasc Imaging. 2014;7:1185–94. 25440592 10.1016/j.jcmg.2014.07.018

[9] Florescu DR, Muraru D, Florescu C, Volpato V, Caravita S, Perger E, et al. Right heart chambers geometry and function in patients with the atrial and the ventricular phenotypes of functional tricuspid regurgitation. Eur Heart J Cardiovasc Imaging. 2022;23:930–40. 34747460 10.1093/ehjci/jeab211

[10] McMurray JJV, Solomon SD, Inzucchi SE, Køber L, Kosiborod MN, Martinez FA, et al. Dapagliflozin in patients with heart failure and reduced ejection fraction. N Engl J Med. 2019;381:1995–2008. 31535829 10.1056/NEJMoa1911303

[11] Packer M, Anker SD, Butler J, Filippatos G, Pocock SJ, Carson P, et al. Cardiovascular and renal outcomes with Empagliflozin in heart failure. N Engl J Med. 2020;383:1413–24. 32865377 10.1056/NEJMoa2022190

[12] Anker SD, Butler J, Filippatos G, Ferreira JP, Bocchi E, Böhm M, et al. Empagliflozin in heart failure with a preserved ejection fraction. N Engl J Med. 2021;385:1451–61. 34449189 10.1056/NEJMoa2107038

[13] Solomon SD, McMurray JJV, Claggett B, de Boer RA, DeMets D, Hernandez AF, et al. Dapagliflozin in heart failure with mildly reduced or preserved ejection fraction. N Engl J Med. 2022;387:1089–98. 36027570 10.1056/NEJMoa2206286

[14] Nootens M, Kaufmann E, Rector T, Toher C, Judd D, Francis GS, et al. Neurohormonal activation in patients with right ventricular failure from pulmonary hypertension: relation to hemodynamic variables and endothelin levels. J Am Coll Cardiol. 1995;26:1581–5. 7594089 10.1016/0735-1097(95)00399-1

[15] Shin Y, Kim KW, Lee AJ, Sung YS, Ahn S, Koo JH, et al. A good practice-compliant clinical trial imaging management system for multicenter clinical trials: development and validation study. JMIR Med Inform. 2019;7:e14310. 31471962 10.2196/14310 PMC6743263

[16] Lee JW, Hur JH, Yang DH, Lee BY, Im DJ, Hong SJ, et al. Guidelines for cardiovascular magnetic resonance imaging from the Korean Society of Cardiovascular Imaging: part 2: interpretation of cine, flow, and angiography data. Korean J Radiol. 2019;20:1477–90. 31606953 10.3348/kjr.2019.0407 PMC6791819

[17] Lang RM, Badano LP, Mor-Avi V, Afilalo J, Armstrong A, Ernande L, et al. Recommendations for cardiac chamber quantification by echocardiography in adults: an update from the American Society of Echocardiography and the European Association of Cardiovascular Imaging. J Am Soc Echocardiogr. 2015;28:1-39.e14. 25559473 10.1016/j.echo.2014.10.003

[18] Rudski LG, Lai WW, Afilalo J, Hua L, Handschumacher MD, Chandrasekaran K, et al. Guidelines for the echocardiographic assessment of the right heart in adults: a report from the American Society of Echocardiography endorsed by the European Association of Echocardiography, a registered branch of the European Society of Cardiology, and the Canadian Society of Echocardiography. J Am Soc Echocardiogr. 2010;23:685–713. 20620859 10.1016/j.echo.2010.05.010

[19] Zoghbi WA, Adams D, Bonow RO, Enriquez-Sarano M, Foster E, Grayburn PA, et al. Recommendations for noninvasive evaluation of native valvular regurgitation: a report from the American Society of Echocardiography developed in collaboration with the Society for Cardiovascular Magnetic Resonance. J Am Soc Echocardiogr. 2017;30:303–71. 28314623 10.1016/j.echo.2017.01.007

[20] Kim HK, Kim YJ, Park EA, Bae JS, Lee W, Kim KH, et al. Assessment of haemodynamic effects of surgical correction for severe functional tricuspid regurgitation: cardiac magnetic resonance imaging study. Eur Heart J. 2010;31:1520–8. 20233787 10.1093/eurheartj/ehq063

[21] Hahn RT, Lindenfeld J, Böhm M, Edelmann F, Lund LH, Lurz P, et al. Tricuspid regurgitation in patients with heart failure and preserved ejection fraction: JACC State-of-the-Art Review. J Am Coll Cardiol. 2024;84:195–212. 38960514 10.1016/j.jacc.2024.04.047

[22] Sorajja P, Whisenant B, Hamid N, Naik H, Makkar R, Tadros P, et al. Transcatheter repair for patients with tricuspid regurgitation. N Engl J Med. 2023;388:1833–42. 36876753 10.1056/NEJMoa2300525

[23] Hahn RT, Makkar R, Thourani VH, Makar M, Sharma RP, Haeffele C, et al. Transcatheter valve replacement in severe tricuspid regurgitation. N Engl J Med. 2025;392:115–26. 39475399 10.1056/NEJMoa2401918

[24] Hahn RT, Thomas JD, Khalique OK, Cavalcante JL, Praz F, Zoghbi WA. Imaging assessment of tricuspid regurgitation severity. JACC Cardiovasc Imaging. 2019;12:469–90. 30846122 10.1016/j.jcmg.2018.07.033

[25] Kang DH, Park SJ, Shin SH, Hong GR, Lee S, Kim MS, et al. Angiotensin receptor neprilysin inhibitor for functional mitral regurgitation. Circulation. 2019;139:1354–65. 30586756 10.1161/CIRCULATIONAHA.118.037077

[26] Kang DH, Park SJ, Shin SH, Hwang IC, Yoon YE, Kim HK, et al. Ertugliflozin for functional mitral regurgitation associated with heart failure: EFFORT trial. Circulation. 2024;149:1865–74. 38690659 10.1161/CIRCULATIONAHA.124.069144

[27] O’Gara PT, Mack MJ. Secondary mitral regurgitation. N Engl J Med. 2020;383:1458–67. 33027570 10.1056/NEJMcp1903331

